# Association of Race and Socioeconomic Status With Colorectal Cancer Screening, Colorectal Cancer Risk, and Mortality in Southern US Adults

**DOI:** 10.1001/jamanetworkopen.2019.17995

**Published:** 2019-12-20

**Authors:** Shaneda Warren Andersen, William J. Blot, Loren Lipworth, Mark Steinwandel, Harvey J. Murff, Wei Zheng

**Affiliations:** 1 Vanderbilt Epidemiology Center, Vanderbilt-Ingram Cancer Center, Division of Epidemiology, Department of Medicine, Vanderbilt University School of Medicine, Nashville, Tennessee; 2 Department of Population Health Sciences, School of Medicine and Public Health, University of Wisconsin, Madison; 3 University of Wisconsin Carbone Cancer Center, Madison; 4 International Epidemiology Field Station, Vanderbilt Institute for Clinical and Translational Research, Nashville, Tennessee; 5 Department of Medicine, Vanderbilt University Medical Center, Nashville, Tennessee

## Abstract

**Importance:**

Colorectal cancer (CRC) screening is rarely studied in populations who may face additional barriers to participate in cancer screening, such as African American individuals and individuals with low socioeconomic status (SES).

**Objective:**

To examine the associations of CRC screening and modalities with CRC incidence and mortality by race and SES.

**Design, Setting, and Participants:**

This cohort study used data from the Southern Community Cohort Study, which enrolled more than 85 000 participants from community health centers or stratified random sampling of the general population in 12 states in the southeastern United States. The present study included data from cohort members who were eligible for CRC screening as recommended by expert organizations based on age and family history. Participants completed questionnaires from 2002 to 2009 and were contacted again from 2008 to 2012. Linkages to state cancer registries and the National Death Index as of December 31, 2016, identified incident CRC and vital status. Data analysis was performed from January 1, 2018, to October 30, 2019.

**Main Outcomes and Measures:**

Incident CRC (n = 632) and mortality (n = 10 003). Cox proportional hazards regression models evaluated associations between screening modalities and CRC risk and mortality. Information on fecal occult blood test use was only obtained on the follow-up questionnaire. Self-identified race was measured as African American/black, white, or other, and SES was defined by household income.

**Results:**

This study included 47 596 participants (median baseline age, 54 years [interquartile range, 10 years]; 32 185 [67.6%] African American; 28 884 [60.7%] female; and 26 075 [54.8%] with household income <$15 000). A total of 24 432 participants (63.9%) had never undergone CRC testing at baseline. The CRC testing assessed at baseline and follow-up interviews was associated with significant CRC risk reduction (hazard ratio [HR], 0.55; 95% CI, 0.44-0.70 for ever colonoscopy at baseline). Results were similar in analyses stratified by race (African American: HR, 0.65; 95% CI, 0.50-0.85; white: HR, 0.44; 95% CI, 0.27-0.70) and household income (<$15 000: HR, 0.63; 95% CI, 0.46-0.86, ≥$15 000: HR, 0.49; 95% CI, 0.35-0.69). Ever sigmoidoscopy at baseline was associated with CRC risk reduction (HR, 0.66; 95% CI, 0.51-0.87), and undergoing fecal occult blood test in the interval between baseline and follow-up interview was associated with CRC risk reduction (HR, 0.75; 95% CI, 0.57-0.98). Inverse associations were also observed between CRC mortality and receipt of colonoscopy (HR for women, 0.39; 95% CI, 0.21-0.73; HR for men, 0.69; 95% CI, 0.40-1.18) and sigmoidoscopy (HR for women, 0.37; 95% CI, 0.16-0.85; HR for men, 0.82; 95% CI, 0.46-1.47); however, the association did not extend to fecal occult blood test (HR for women, 1.02; 95% CI, 0.62-1.70; HR for men, 1.03; 95% CI, 0.55-1.93).

**Conclusions and Relevance:**

In this study, CRC test rates were low among African American individuals and those with low SES. The findings suggest that screening, particularly with colonoscopy, is significantly associated with reduced risk of CRC and mortality. The CRC disparities experienced by individuals with low SES and African American individuals may be lessened by improving access to and uptake of CRC screening.

## Key Points

**Question:**

What are the associations between colorectal cancer screening modalities with colorectal cancer risk and mortality among African American individuals and individuals with low socioeconomic status, who more often face barriers to screening and experience colorectal cancer health disparities?

**Findings:**

In this cohort study of 47 596 adults, colorectal cancer screening was significantly associated with reduced colorectal cancer risk and mortality. Use of colonoscopy was associated with a 45% reduced incidence of colorectal cancer, use of sigmoidoscopy with a 34% reduced incidence, and stool-based tests with a 25% reduced incidence compared with no screening.

**Meaning:**

The large colorectal cancer disparities experienced by individuals with low socioeconomic status and African American individuals may be lessened by improving access to and uptake of colorectal cancer screening.

## Teaser

This cohort study examines the associations of colorectal cancer screening and modalities with colorectal cancer incidence and mortality by race and socioeconomic status among southern US adults.

## Introduction

Colorectal cancer (CRC) is the fourth most commonly diagnosed cancer in the United States and second-leading cause of cancer death.^1^ Colorectal cancer outcomes vary by race and socioeconomic status (SES), with African American individuals and individuals with low SES having higher incidence and mortality than other groups. Most CRCs arise from noncancerous, adenomatous polyps.^2^ Colorectal cancer screening programs aim to intervene in the cancer progression process by the detection and subsequent removal of polyps before they progress to cancer and by early detection of CRCs in asymptomatic individuals. The importance of CRC screening by visual- and stool-based screening modalities for reducing CRC incidence and mortality has been demonstrated in randomized clinical trials and cohort studies.^3,4,5,6,7,8^ Cohort studies provide opportunities to evaluate the effectiveness of CRC screening in targeted populations. A previous cohort study^5^ of data from follow-up of nurses and other health care professionals reported 40% to 56% lower CRC incidence among participants screened through lower endoscopy (colonoscopy or sigmoidoscopy) compared with participants who had never undergone lower endoscopy screening, and a 68% reduction in colorectal cancer mortality among participants who had undergone screening colonoscopy. Stool-based screening programs, such as fecal occult blood tests (FOBTs) and fecal immunochemical tests, have also been shown to be effective for detecting colorectal polyps and cancers.^8^ Randomized clinical trials of stool-based screening modalities have found reduced CRC incidence and mortality among patients with abnormal stool-based screening findings who underwent diagnostic colonoscopy and polypectomy.^8,9^

The effectiveness of CRC screening has been rarely studied in African American individuals or populations with low SES, who may face additional barriers to participate in cancer screening or receive less than optimal medical care. Specifically, difference in screening participation is hypothesized to contribute to disparities in CRC outcomes.^10,11,12^ Barriers that may contribute to the effectiveness of CRC screening among African American patients or patients with low SES include inadequate insurance coverage, lack of a medical home, less access to financial resources, less access to transportation, and schedule availability.^13,14^ In addition, African American individuals report lower quality of health care and may be subject to implicit racial bias of their health care practitioners.^15^ The Southern Community Cohort Study (SCCS) is a landmark prospective cohort study designed to ensure the cohort’s makeup is mostly African American and of low SES to address questions related to cancer disparities associated with SES and race. The SCCS cohort provided a unique opportunity to investigate CRC test patterns and associations with CRC risk and mortality in underserved racial and socioeconomic populations. We evaluated the associations between CRC test modalities and incident CRC and mortality.

## Methods

### Study Population

This cohort study used data from the SCCS (detailed descriptions of the prospective SCCS have been previously published).^16,17^ In brief, the SCCS enrolled more than 85 000 adult participants from 2002 to 2009 in 12 states in the southeastern United States. Participants were aged 40 to 79 years at enrollment and English speaking. Most participants were enrolled at community health centers, which are health care organizations that primarily work with medically underserved populations. The remaining cohort participants were enrolled using an identical mailed questionnaire sent to stratified random samples of residents in the same 12 states. At baseline study entry, all participants completed questionnaires to obtain information on cancer screening participation, lifestyle habits, personal and familial medical history, and demographic characteristics, including self-identified race and sex. Participants self-identified their race according to categories defined by the investigators. The SCCS was approved by institutional review boards at Vanderbilt University Medical Center and Meharry Medical College. All participants provided written informed consent, and data were deidentified. This study followed the Strengthening the Reporting of Observational Studies in Epidemiology (STROBE) reporting guideline.

Ascertainment of information on the cohort subsequent to baseline enrollment has been obtained through passive and active methods. Participants were contacted again from 2008 to 2012 to obtain updated information on health history and lifestyle exposures via telephone or mailed paper questionnaire. A total of 34 516 participants (72.5%) in this study completed the follow-up interview. The median time between baseline and follow-up interview was 4.7 years (interquartile range [IQR], 1.9 years). Linkages to state cancer registries and the National Death Index through December 31, 2016, acquired information on site-specific cancer incidence and cause-specific mortality. Incident colon or rectal cancer were defined by *International Classification of Diseases for Oncology* codes C180 to C189, C199, and C209 and CRC mortality by *International Statistical Classification of Diseases and Related Health Problems, Tenth Revision* codes C18 to C20.

### Analytical Data Set: Participant Eligibility Information

Participants eligible for inclusion in the study were without diagnosis of any cancer (not including nonmelanoma skin cancer) or colorectal adenoma at or before study entry. Participants also must have been eligible for CRC screening using the screening recommendations for the period of enrollment into the SCCS (2002-2009) from the US Preventive Services Task Force^18,19^ or the joint recommendation from the American Cancer Society, the US Multi-Society Task Force on CRC, and the American College of Radiology.^20,21^ The CRC screening recommendations applied to adults 50 years or older at study entry and adults 40 years or older at study entry who had a family history of CRC in a first-degree relative. A second group of participants included those who became eligible for inclusion at the follow-up interview by meeting the eligibility criterion of age of 50 years or older or a family history of CRC at the follow-up interview. We identified 38 211 participants eligible for CRC screening at study enrollment and 47 596 participants eligible for CRC screening at their follow-up interview.

### Exposure Assessment: CRC Test Participation Before Baseline Enrollment and Between Baseline and Follow-up Interviews

We examined the associations between CRC outcomes and use of the following CRC test modalities: colonoscopy, sigmoidoscopy, or stool-based screening. At baseline and follow-up interviews, participants were asked about their use of colonoscopy or sigmoidoscopy. In the calendar period between baseline and follow-up interviews, use of stool-based screening modalities became more common in the United States; thus, during the follow-up interview, participants were asked if they had an FOBT since joining the study.

We conducted stratified analysis during which we categorized participants into groups by available follow-up interview information, CRC status, and vital status (eFigure in the Supplement). Associations with CRC incidence were first evaluated by assessing associations between ever participation in CRC screening at baseline and follow-up interviews, and analyses were separately conducted to quantify the associations of colonoscopy, sigmoidoscopy, and FOBT with CRC incidence and mortality.

### Statistical Analysis

Frequency distributions of participant characteristics were tabulated by CRC diagnosis and vital status and by CRC test participation. We evaluated the association between race and participation in colonoscopy using multivariable logistic regression. Hazard ratios (HRs) and 95% CIs were estimated using Cox proportional hazards regression models for the associations between CRC test variables and CRC incidence, CRC mortality, and all-cause mortality. We evaluated associations with non-CRC mortality and found similar results as analyses for all-cause mortality; thus, we present results only for all-cause mortality. Age was used as the time scale. In analyses using data from the baseline interview, entry time was defined as age at study entry. For CRC risk models, exit time was defined as age at CRC diagnosis, age at loss to follow-up, or December 31, 2016. For mortality models, exit time was defined as age at death, age at loss to follow-up, or December 31, 2016. In analyses in which the exposure was CRC testing in the interval between baseline and follow-up interview, entry time was defined as the participant’s age at the median of the interval between baseline and follow-up interview because we were unable to determine the date at which the participant underwent CRC testing. Participants who contributed no follow-up time were not included in statistical models. When CRC incidence was identified from a participant’s death certificate, the date of CRC onset was set to the date of death (n = 45). Participants with missing cause of death (n = 85) were excluded from CRC mortality analyses. We evaluated the proportional hazards assumption graphically and considered it met.

Statistical models included the following variables selected a priori as potential confounders because of known and hypothesized associations with CRC screening and CRC outcomes: self-identified race (African American, white, or other), sex, enrollment source (community health center or non–community health center), health insurance status (yes or no), annual household income (<$15 000, $15 000-24 999, $25 000-49 999, or ≥$50 000), educational level (<9 years, 9-11 years, high school, some college, or college graduate and beyond), body mass index (<18.5, 18.5-24.9, 25.0-29.9, 30.0-34.9, or ≥35.0 [calculated as weight in kilograms divided by square of height in meters]), smoking status (never, former, or current), alcohol intake (women: 0, ≤1 drink, or >1 drink per day; men: 0, ≤2, or >2 drinks per day), and family history of CRC diagnosis in a first-degree relative (yes, no, or unknown). Missing covariate data (<2% of the variable) were set to sex- and race-specific medians or modes.

We assessed the possibility of differences in the association between CRC screening and CRC outcomes by race, sex, and household income. Possible interactions between CRC testing and factors of interest were assessed by likelihood ratio tests to compare main outcomes models with and without the addition of cross-product terms. Statistical analyses were performed using SAS statistical software, version 9.4 (SAS Institute Inc) from January 1, 2018, to October 30, 2019. A 2-sided *P* < .05 was considered to be statistically significant.

## Results

This study included 47 596 participants (median baseline age, 54 years [IQR, 10.0 years]; 32 185 [67.6%] African American; 28 884 [60.7%] female; and 26 075 [54.8%] with household income <$15 000). During 478 275 person-years of follow-up, we observed 632 incident CRC cases and 204 deaths from CRC. The mean (SD) follow-up time from baseline to end of study or death was 10.6 (3.0) years (median, 11.3 years; IQR, 3.7 years). Participants who subsequently developed CRC were older and more likely to be African American, have lower household income, and have a history of smoking cigarettes than those who remained CRC free at the end of the follow-up (Table 1). The mean (SD) age at CRC diagnosis in the study was 63.3 (8.2) years, with 12 patients (1.9%) younger than 50 years at diagnosis. Patients with CRC who died during the study were more likely than patients alive at the end of study to be older at baseline, to be male, to have less educational attainment, to have a lower household income, and to report current smoking at baseline. Patients who died during the study were also less likely to report a family history of CRC and were more often diagnosed with a more advanced-stage cancer than the patients alive at the end of study.

**Table 1.** Selected Baseline Characteristics of Southern Community Cohort Study Participants, 2002-2009, by CRC Incidence and Vital Status^a^

Characteristic	Cohort (N = 47 596)	Patients With Incident CRC			
		All (n = 632)	Alive (n = 317)	Deceased	
				All Cause (n = 315)	CRC (n = 204)
Age, y					
<50	11 100 (23.3)	88 (13.9)	56 (17.7)	32 (10.2)	25 (12.3)
50-54	14 216 (29.9)	168 (26.6)	90 (28.4)	78 (24.8)	50 (24.5)
55-59	9894 (20.8)	150 (23.7)	73 (23.0)	77 (24.4)	50 (24.5)
60-64	6315 (13.3)	108 (17.1)	60 (18.9)	48 (15.2)	32 (15.7)
≥65	6071 (12.8)	118 (18.7)	38 (12.0)	80 (25.4)	47 (23.0)
Male	18 712 (39.3)	253 (40.7)	115 (36.3)	142 (45.1)	93 (45.6)
Enrollment source					
Community health center	40 878 (85.9)	556 (88.0)	273 (86.1)	283 (89.8)	181 (88.7)
General population	6718 (14.1)	76 (12.0)	44 (13.9)	32 (10.2)	23 (11.3)
Race					
African American	32 185 (67.6)	470 (74.4)	235 (74.1)	235 (74.6)	152 (74.5)
White	13 381 (28.1)	142 (22.5)	69 (21.8)	73 (23.2)	49 (24.0)
Other	2030 (4.3)	20 (3.2)	13 (4.1)	7 (2.2)	3 (1.5)
Educational attainment					
Less than high school	14 401 (30.3)	216 (34.2)	95 (30.0)	121 (38.4)	72 (35.3)
High school	14 807 (31.1)	200 (31.6)	103 (32.5)	97 (30.8)	59 (28.9)
More than high school	18 141 (38.1)	212 (33.5)	117 (36.9)	95 (30.2)	73 (35.8)
Annual household income, $					
<15 000	26 075 (54.8)	376 (59.5)	174 (54.9)	202 (64.1)	126 (61.8)
15 000-49 999	16 339 (34.3)	199 (31.5)	103 (32.5)	96 (30.5)	65 (31.9)
≥50 000	4470 (9.4)	46 (7.3)	31 (9.8)	15 (4.8)	12 (5.9)
Insurance, yes	29 889 (62.8)	374 (59.2)	177 (55.8)	197 (62.5)	124 (60.8)
Family history of CRC	4194 (8.8)	60 (9.5)	36 (11.4)	24 (7.6)	18 (8.8)
Obesity, BMI ≥30	21 387 (44.9)	283 (44.8)	148 (46.7)	135 (42.9)	89 (43.6)
Smoking status					
Never	18 099 (38.0)	250 (39.6)	131 (41.3)	119 (37.8)	81 (39.7)
Former	11 841 (24.9)	177 (28.0)	91 (28.7)	86 (27.3)	53 (26.0)
Current	17 326 (36.4)	202 (32.0)	93 (29.3)	109 (34.6)	69 (33.8)
Nonconsumers and moderate consumers of alcohol^b^	39 647 (83.3)	551 (87.2)	277 (87.4)	274 (87.0)	172 (84.3)
Physical activity, median (IQR), MET-h/d	16.5 (20.1)	16.6 (19.3)	17.7 (21.0)	15.2 (18.3)	15.2 (19.3)
Sedentary time, median (IQR), h	8.3 (6.5)	8.0 (5.5)	8.0 (6.1)	7.5 (5.5)	7.5 (5.5)
Age at diagnosis, median (IQR), y	NA	62 (12)	62 (10)	63 (13)	63 (13)
Anatomical site^c^					
Colon	NA	481 (76.1)	260 (82.0)	221 (70.2)	139 (68.1)
Rectum	NA	146 (23.1)	57 (18.0)	49 (15.6)	27 (13.2)
Stage of tumor at diagnosis					
0	NA	16 (2.5)	12 (3.8)	4 (1.3)	NA
1	NA	101 (16.0)	81 (25.6)	20 (6.3)	7 (3.4)
2	NA	96 (15.2)	58 (18.3)	38 (12.1)	11 (5.4)
3	NA	109 (17.2)	66 (20.8)	43 (13.7)	25 (12.3)
4	NA	108 (17.1)	16 (5.0)	92 (29.2)	75 (36.8)
Missing	NA	202 (32.0)	84 (26.5)	118 (37.5)	86 (42.2)

Abbreviations: BMI, body mass index (calculated as weight in kilograms divided by height in meters squared); CRC, colorectal cancer; IQR, interquartile range; MET, metabolic equivalent; NA, not applicable.

^a^ Data are presented as number (percentage) of participants unless otherwise indicated. Individuals with missing data were not included in this analysis.

^b^ Nonconsumption to moderate alcohol consumption was defined as alcohol intake of 1 drink per day or less for women or 2 drinks per day or less for men.

^c^ Counts include cases identified from state cancer registries. Participants who were diagnosed with colon and rectal cancer are reported in the data summary for colon cancer.

Of the participants eligible for screening at the baseline interview, 13 371 (35.0%) reported undergoing CRC tests at their baseline interview; however, the rate varied by modality, SES, smoking status, and race (Figure and eTable 1 and eTable 2 in the Supplement). At baseline, colonoscopy was more commonly used than sigmoidoscopy (10 413 [27.4%] vs 5978 [15.7%]), and 3020 participants (7.9%) had used both modalities. Participants enrolled in the study at a community health service were less likely to have undergone lower endoscopy than participants enrolled through the general population (10 622 [32.4%] vs 2749 [50.2%]). The CRC test rates were highest among participants with an annual household income of $50 000 or greater (1989 [55.5%]). The rate of lower endoscopy before baseline was particularly low for individuals who were uninsured (2926 [22.0%]) or current smokers (3359 [25.5%]).

**Figure.**
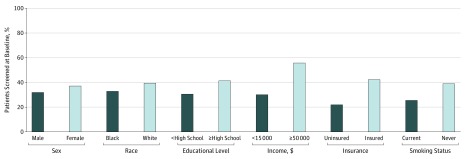
Distribution of Ever Participation in Lower Endoscopy at the Baseline Interview Colorectal cancer test participation among participants not diagnosed with incident colorectal cancer during the study period and who were eligible for colorectal cancer screening at the baseline interview. All comparisons were significant at *P* < .05. Additional baseline characteristics by colorectal cancer test participation are given in eTable 1 and eTable 2 in the Supplement.

Similar to a previous report^22^ using initial SCCS data, at the baseline interview, colonoscopy rates were lower among African American individuals than among white individuals (6230 [24.5%] vs 3643 [32.7%], *P* < .001), but sigmoidoscopy rates were similar (3928 [15.5%] vs 1764 [15.8%], *P* = .54). Age may have been associated with the observed racial/ethnic differences in colonoscopy rates because test use increased with age at entry and, on average, white participants were slightly older at baseline than African American participants (median age, 57 years [IQR, 9 years] vs 55 years [IQR, 9 years]). However, the odds of colonoscopy at baseline were lower among African American participants than among white participants even with adjustment for age (odds ratio, 0.71; 95% CI, 0.68-0.75). In the interval between baseline and the follow-up interview, colonoscopy (12 047 [36.1%]) and FOBT (12 039 [35.0%]) were more commonly reported than sigmoidoscopy (3215 [9.4%]), and the frequency did not vary by race.

Ever colonoscopy at baseline was associated with lower CRC risk (HR, 0.55; 95% CI, 0.44-0.70) and ever sigmoidoscopy at baseline was associated with CRC risk reduction (HR, 0.66; 95% CI, 0.51-0.87) (Table 2). Results were similar in analyses stratified by race (African American: HR, 0.65; 95% CI, 0.50-0.85; white: HR, 0.44; 95% CI, 0.27-0.70) and household income (<$15 000: HR, 0.63; 95% CI, 0.46-0.86, ≥$15 000: HR, 0.49; 95% CI, 0.35-0.69). Similarly, the inverse association between lower endoscopy before the baseline and CRC risk was consistent in all strata in analyses that were stratified by sex (men: HR, 0.64; 95% CI, 0.47-0.88; women: HR, 0.48; 95% CI, 0.36-0.63; *P* = .09 for interaction), household income (<$15 000: HR, 0.59; 95% CI, 0.45-0.78; ≥$15 000: HR, 0.51; 95% CI, 0.37-0.69; *P* = .39 for interaction), and race (white: HR, 0.48; 95% CI, 0.31-0.72; African American: HR, 0.59; 95% CI, 0.46-0.75; *P* = .34 for interaction) (eTable 3 in the Supplement presents incidence results for African American participants). The inverse associations between lower endoscopy and CRC risk were also observed in analyses stratified by the availability of follow-up interview data; among the participants eligible at baseline interview, lower endoscopy before baseline was associated with a 46% (95% CI, 33%-56%) decreased risk of CRC, and among the participants eligible at the follow-up interview, participation in lower endoscopy in the interval between baseline and the follow-up interview was associated with a 48% (95% CI, 31%-61%) decreased risk of CRC.

**Table 2.** Associations Between Lower Endoscopy and Colorectal Cancer Incidence by Timing of CRC Testing Among Southern Community Cohort Study Participants Eligible for CRC Screening at the Baseline Interview

CRC Test Participation	Lower Endoscopy			Colonoscopy			Sigmoidoscopy		
	Total Cohort, No.	Patients With Incident CRC, No.	HR (95% CI)^a^	Total Cohort, No.	Patients With Incident CRC, No.	HR (95% CI)^a^	Total Cohort, No.	Patients With Incident CRC, No.	HR (95% CI)^a^
All participants									
Never tested before baseline	24 432	418	1 [Reference]	27 382	457	1 [Reference]	31 841	483	1 [Reference]
Ever tested before baseline	13 371	135	0.54 (0.44-0.67)	10 413	97	0.55 (0.44-0.70)	5978	69	0.66 (0.51-0.87)
Patients diagnosed before the follow-up interview									
Never tested before baseline	24 275	261	1 [Reference]	27 208	283	1 [Reference]	31 654	296	1 [Reference]
Ever tested before baseline	13 311	75	0.52 (0.39-0.69)	10 370	54	0.55 (0.40-0.75)	5948	39	0.63 (0.43-0.90)
Patients diagnosed after the follow-up interview^b^									
Never tested before follow-up interview	9013	107	1 [Reference]	10 263	118	1 [Reference]	16 005	155	1 [Reference]
Tested before baseline only	4458	32	0.54 (0.36-0.81)	3562	23	0.53 (0.33-0.83)	3473	25	0.65 (0.42-0.99)
Tested between baseline and follow-up interview only	4806	34	0.52 (0.35-0.77)	5542	39	0.53 (0.37-0.77)	1762	10	0.49 (0.26-0.94)
Tested both before baseline and between baseline and follow-up interview	5245	28	0.37 (0.24-0.57)	4155	20	0.36 (0.22-0.59)	754	5	0.55 (0.22-1.34)

Abbreviations: CRC, colorectal cancer; HR, hazard ratio.

^a^ Analyses include participants eligible for CRC screening at the baseline interview based on age and family history criteria. Analyses were adjusted for enrollment source, race, sex, health insurance status, smoking status, educational level, income, alcohol intake, body mass index, and family history of CRC. Statistical models included variables for unknown CRC testing status at baseline or the follow-up interview.

^b^ Participants included in the analysis must have completed the follow-up interview.

Analysis of the association between FOBT screening in the interval between baseline and the follow-up interview revealed an inverse association (HR, 0.75; 95% CI, 0.57-0.98), although the association was less prominent than the association observed for colonoscopy (HR, 0.53; 95% CI, 0.40-0.71) and sigmoidoscopy (HR, 0.53; 95% CI, 0.32-0.86) for the same interval (Table 3).

**Table 3.** Associations Between CRC Testing Between the Baseline and Follow-up Interviews With CRC Incidence and Mortality Among Southern Community Cohort Study Participants Eligible for CRC Screening at the Follow-up Interview

Variable	Any Test Modality			Colonoscopy			Sigmoidoscopy			Fecal Occult Blood Test		
	Cohort, No.	Events, No.	HR (95% CI)^a^	Cohort, No.	Events, No.	HR (95% CI)^a^	Cohort, No.	Events, No.	HR (95% CI)^a^	Cohort, No.	Events, No.	HR (95% CI)^a^
**CRC Incidence**												
Not tested between the baseline and follow-up interviews	13 968	129	1 [Reference]	19 663	179	1 [Reference]	26 765	227	1 [Reference]	18 933	159	1 [Reference]
Tested between the baseline and follow-up interviews	17 907	118	0.62 (0.48-0.80)	12 407	68	0.53 (0.40-0.71)	3215	17	0.53 (0.32-0.86)	12 039	85	0.75 (0.57-0.98)
**CRC Mortality**												
Women												
Not tested between the baseline and follow-up interviews	8967	28	1 [Reference]	12 624	38	1 [Reference]	17 412	54	1 [Reference]	12 420	37	1 [Reference]
Tested between the baseline and follow-up interview	12 006	35	0.85 (0.51-1.42)	8492	25	0.94 (0.56-1.57)	2111	8	1.06 (0.50-2.24)	7794	26	1.02 (0.62-1.70)
Men												
Not tested between the baseline and follow-up interviews	4993	14	1 [Reference]	7036	20	1 [Reference]	9403	31	1 [Reference]	6547	23	1 [Reference]
Tested between the baseline and follow-up interviews	6025	28	1.41 (0.72-2.75)	4035	22	1.74 (0.92-3.29)	1151	11	2.31 (1.14-4.70)	4318	19	1.03 (0.55-1.93)

Abbreviations: CRC, colorectal cancer; HR, hazard ratio.

^a^ Analyses include participants eligible for CRC screening at the follow-up interview based on age and family history criteria who completed the follow-up interview and were not diagnosed with CRC before the follow-up interview. Analyses were adjusted for enrollment source, race, sex, health insurance status, smoking status, educational level, income, alcohol intake, body mass index, and family history of CRC. Statistical models included variables for unknown CRC testing status at baseline or the follow-up interview.

A high mortality rate was found among participants diagnosed with incident CRC; 315 participants (49.8%) died during the study period, with most deaths among those diagnosed with stage IV disease or missing stage at diagnosis (Table 1). The association between lower endoscopy before baseline and CRC mortality did not vary by household income or race (eTable 4 in the Supplement). However, we observed sex-specific differences in the association between lower endoscopy and CRC mortality (*P* = .003 for interaction). Women who had lower endoscopy before baseline had a reduced risk of CRC mortality (HR, 0.34; 95% CI, 0.19-0.60), and the inverse association between lower endoscopy before baseline and CRC mortality was consistent in analyses stratified by the availability of follow-up interview data (Table 4). Among men, ever use of lower endoscopy was not associated with CRC mortality (HR, 0.70; 95% CI, 0.43-1.14). When the data were stratified by the availability of follow-up interview data, the association became more apparently null in all subgroups, likely because of small sample size and the changing composition of the reference group.

**Table 4.** Associations Between Lower Endoscopy and CRC Mortality by Timing of CRC Testing and Sex Among Southern Community Cohort Study Participants Eligible for CRC Screening at the Baseline Interview

Participants	Lower Endoscopy			Colonoscopy			Sigmoidoscopy		
	Cohort, No.	Deaths, No.	HR (95% CI)^a^	Cohort, No.	Deaths, No.	HR (95% CI)^a^	Cohort, No.	Deaths, No.	HR (95% CI)^a^
**Women**									
All participants									
Never tested before baseline	14 135	75	1 [Reference]	15 882	79	1 [Reference]	19 012	84	1 [Reference]
Ever tested before baseline	8454	16	0.34 (0.19-0.60)	6705	12	0.39 (0.21-0.73)	3582	7	0.37 (0.16-0.85)
Participants without follow-up interview data									
Never tested before baseline	4174	38	1 [Reference]	4639	41	1 [Reference]	5259	39	1 [Reference]
Ever tested before baseline	1963	5	0.24 (0.09-0.69)	1496	2	0.17 (0.04-0.71)	888	4	0.48 (0.15-1.56)
Participants with follow-up interview data^b^									
Never tested before the follow-up interview	5843	22	1 [Reference]	6652	22	1 [Reference]	10 621	37	1 [Reference]
Tested before baseline only	2947	6	0.51 (0.20-1.26)	2379	6	0.78 (0.31-1.94)	2224	3	0.35 (0.11-1.13)
Tested between the baseline and follow-up interviews only	3309	14	0.97 (0.49-1.91)	3791	15	1.06 (0.54-2.05)	1167	6	1.18 (0.49-2.81)
Tested before baseline and between the baseline and follow-up interviews	3544	5	0.34 (0.12-0.90)	2830	4	0.43 (0.14-1.27)	470	0	NA
**Men**									
All participants									
Never tested before baseline	10 245	61	1 [Reference]	11 440	69	1 [Reference]	12 768	73	1 [Reference]
Ever tested before baseline	4895	26	0.70 (0.43-1.14)	3694	18	0.69 (0.40-1.18)	2383	14	0.82 (0.46-1.47)
Participants without follow-up interview data									
Never tested before baseline	5049	32	1 [Reference]	5539	34	1 [Reference]	5889	41	1 [Reference]
Ever tested before baseline	1663	15	1.10 (0.58-2.09)	1173	13	1.54 (0.79-3.00)	838	6	0.76 (0.32-1.83)
Participants with follow-up interview data^b^									
Never tested before the follow-up interview	3171	9	1 [Reference]	3615	14	1 [Reference]	5425	19	1 [Reference]
Tested before baseline only	1512	6	1.19 (0.41-3.44)	1182	2	0.37 (0.08-1.67)	1260	6	1.11 (0.43-2.86)
Tested between the baseline and follow-up interviews only	1581	17	3.70 (1.62-8.46)	1839	18	2.38 (1.16-4.91)	631	9	3.45 (1.53-7.75)
Tested both before baseline and between the baseline and follow-up interviews	1720	5	0.81 (0.26-2.54)	1339	3	0.46 (0.13-1.67)	285	2	1.70 (0.38-7.53)

Abbreviations: CRC, colorectal cancer; HR, hazard ratio; NA, not applicable.

^a^ Analyses include participants eligible for CRC screening at the baseline interview based on age and family history criteria. Models were adjusted for enrollment source, race, health insurance status, smoking status, educational level, income, alcohol intake, body mass index, and family history of CRC. Statistical models included variables for unknown CRC testing status at baseline or the follow-up interview.

^b^ Participants included in the analysis must have completed the follow-up interview.

Among participants who were only screened between baseline and the follow-up interview, there was no association between lower endoscopy use and death from CRC among women (HR, 0.97; 95% CI, 0.49-1.91), whereas there was an association between lower endoscopy and increased CRC mortality among men (HR, 3.70; 95% CI, 1.62-8.46). In the interval between baseline and follow-up interviews, FOBT was not associated with CRC mortality in men or women (Table 3).

We also evaluated associations with all-cause mortality and did not observe outcome modification by sex. Among participants eligible for CRC screening at baseline interview, lower endoscopy before baseline was associated with null or modest increased mortality (eTable 5 in the Supplement). Compared with participants who had never been tested at the follow-up interview, there was reduced all-cause mortality among participants who underwent colonoscopy only in the interval between baseline and the follow-up interview and participants who underwent lower endoscopy before baseline and between baseline and the follow-up interview (eTable 6 in the Supplement).

## Discussion

In this cohort primarily composed of African American individuals and individuals with low SES, CRC test participation was low. At baseline, 63.9% of the cohort had never undergone CRC testing despite meeting criteria for screening recommendations based on age and family history of CRC. For comparison, during the same period, the US national rate of colorectal test use was approximately 50%.^23^ In addition, CRC test rates were lowest among the groups who most often experience adverse CRC outcomes: African American individuals, individuals without health insurance, and those who smoke. Our study results suggest that efforts in public health may improve CRC screening rates with implementation strategies that focus on these high-risk groups and that aim to lower barriers to CRC screening. These barriers include limited access to gastroenterologists to perform screening or diagnostic colonoscopy and cost-prohibitive out-of-pocket expenses.^24^

Although CRC test rates were low, we did observe strong inverse associations between CRC test modalities and CRC incidence. Our results using data from traditionally underserved populations are in line with previous studies^3,4,5,6,7,8^ that found that CRC screening by visual- and stool-based screening modalities was associated with reduced CRC incidence and mortality. Specifically, the US Preventive Services Task Force asserts that the CRC test modalities examined in the present study (colonoscopy, sigmoidoscopy, and FOBT) have differing abilities to detect CRC, but all have been shown to be associated with reduced CRC incidence and mortality in the US general population.^8^ Compared with the cohort data from our study that used information from individuals who primarily have low SES and are African American, data from a cohort that primarily includes individuals with higher SES who are white indicated a similar association of lower endoscopy use and CRC incidence. A report^5^ from the Nurses’ Health Study and the Health Professionals Follow-up Study found that lower endoscopy was associated with reduced CRC incidence (HR, 0.44; 95% CI, 0.38-0.52).

We also observed an inverse association between CRC testing and CRC mortality, although the association was most apparent among women. Limited sample size in our sex-specific analyses may have contributed to wide CIs and lack of statistically significant findings among men. Among men, use of lower endoscopy between baseline and the follow-up interview was associated with increased CRC mortality, raising the possibility that these participants may have undergone lower endoscopy as a diagnostic test rather than as a screening test. Similar to a previous studies,^8^ we did not find consistent associations between CRC testing and all-cause mortality.

Of importance, the risk-lowering benefits of CRC screening associated with CRC risk and mortality did not vary by race or household income. Our findings suggest that increasing uptake of CRC screening among African American individuals may reduce the sizable racial disparity in CRC incidence and mortality. Factors hypothesized to contribute to the racial disparities in CRC outcomes include differences in the prevalence of risk factors^25^ and CRC screening.^10,11,12^ In general, African American individuals, individuals with lower household income, and uninsured individuals are less likely to be concordant with CRC screening guidelines.^22,26,27,28^ In fact, access to and use of health care may play a more important role in CRC racial disparities than other risk factors, as evidenced by data from the Prostate, Lung, Colorectal, and Ovarian (PLCO) Cancer Screening Trial and others.^2,14,29^ For instance, in PLCO Cancer Screening Trial participants randomized to sigmoidoscopy screening, the screening test was equally likely to detect abnormal colorectal findings among African American and white individuals; however, African American participants were less likely than their white counterparts to follow up for additional diagnostic tests after the abnormal sigmoidoscopy findings.^29^ The inability of patients to afford insurance cost sharing and the logistical difficulties associated with transportation and scheduling are hypothesized to contribute to the lower rates of follow-up health care after abnormal CRC screening findings.^2,14,29^

### Strengths and Limitations

This study has a number of strengths. The SCCS is a large, prospective cohort study with systematic follow-up to identify incident CRC diagnoses. The cohort covers an underserved at-risk population that is seldom included in large numbers in other investigations.

This study has limitations. Primarily, we used a crude measure of yes or no to classify screening exposure, instead of determining whether participants received screening under expert-recommended intervals (ie, every 10 years for colonoscopy). In addition, we used self-reported CRC tests instead of the criterion standard of medical records. Prior studies^30,31^ suggest that participants self-report procedures at higher rates than the rates measured via review of health records. However, despite the limitations of potential overreporting of the exposure and lack of information on adherence to screening interval, we were able to detect inverse associations between self-reported CRC tests and CRC outcomes.

Another limitation of the study is the inability to distinguish between lower endoscopies performed for screening vs diagnostic purposes. Our CRC screening exposure variable may include diagnostic tests for individuals already exhibiting symptoms of CRC, which may have attenuated the results in the CRC incidence analysis and contributed to the CRC mortality associations observed with lower endoscopy use between baseline and the follow-up interview among men. A possible future direction of this work may be to incorporate data from health records or health care claims to verify the timing and indication for the colorectal test encounter.

## Conclusions

This study revealed associations between use of lower endoscopy or FOBT and reduced CRC risk and a significant association between lower endoscopy and CRC mortality risk reduction among women. The lowest CRC testing rates were found among individuals who lacked insurance, had low SES, were current smokers, or were African American. Our results may be applicable to other underserved populations, particularly groups with low SES as defined by educational level and income. Promotion of CRC screening targeted at these high-risk groups may improve CRC disparities. In addition, lowering financial barriers and increasing access to CRC screening for the uninsured and underinsured may increase participation in screening and ultimately reduce CRC racial and socioeconomic disparities in incidence and mortality.

## Notes

Supplement. **eTable 1.** Selected Baseline Characteristics by Colorectal Cancer Screening Participation, Among Cohort Members Not Diagnosed With Incident Colorectal Cancer **eTable 2.** Selected Baseline Characteristics by Colorectal Cancer Screening Participation, Among Cohort Members Diagnosed With Incident Colorectal Cancer **eTable 3.** Associations Between Lower Endoscopy and Colorectal Cancer Incidence Stratified by Timing of CRC Screening, Among African American Participants Eligible for Screening at Baseline Interview **eTable 4.** Associations Between Lower Endoscopy and Colorectal Cancer Mortality Stratified by Timing of CRC Screening and Sex, Among African American Participants Eligible for Screening at Baseline Interview **eTable 5.** Associations Between Lower Endoscopy and All-Cause Mortality Stratified by Timing of CRC Screening, Among Participants Eligible for Screening at Baseline Interview **eTable 6.** Associations Between Colorectal Cancer Screening in the Interval Between Baseline and Follow-up Interview With All-Cause Mortality **eFigure.** Study Design
