# Supplementary material for: Association of Race and Socioeconomic Status With Colorectal Cancer Screening, Colorectal Cancer Risk, and Mortality in Southern US Adults
Source: JAMA Netw Open. 2019 Dec 20;2(12):e1917995. doi: 10.1001/jamanetworkopen.2019.17995 (PMC6991213; doi:10.1001/jamanetworkopen.2019.17995)
Supplement: Supplement. — eTable 1. Selected Baseline Characteristics by Colorectal Cancer Screening Participation, Among Cohort Members Not Diagnosed With Incident Colorectal Cancer eTable 2. Selected Baseline Characteristics by Colorectal Cancer Screening Participation, Among Cohort Members Diagnosed With Incident Colorectal Cancer eTable 3. Associations Between Lower Endoscopy and Colorectal Cancer Incidence Stratified by Timing of CRC Screening, Among African American Participants Eligible for Screening at Baseline Interview eTable 4. Associations Between Lower Endoscopy and Colorectal Cancer Mortality Stratified by Timing of CRC Screening and Sex, Among African American Participants Eligible for Screening at Baseline Interview eTable 5. Associations Between Lower Endoscopy and All-Cause Mortality Stratified by Timing of CRC Screening, Among Participants Eligible for Screening at Baseline Interview eTable 6. Associations Between Colorectal Cancer Screening in the Interval Between Baseline and Follow-up Interview With All-Cause Mortality eFigure. Study Design [file jamanetwopen-2-e1917995-s001.pdf]

Supplementary Online Content

Warren Andersen S, Blot WJ, Lipworth L, Steinwandel M, Murff HJ, Zheng W. Association of race and socioeconomic status with colorectal cancer screening, colorectal cancer risk, and mortality in southern US adults. *JAMA Netw Open*. 2019;2(12):e1917995. doi:10.1001/jamanetworkopen.2019.17995

eTable 1. Selected Baseline Characteristics by Colorectal Cancer Screening Participation, Among Cohort Members Not Diagnosed With Incident Colorectal Cancer

eTable 2. Selected Baseline Characteristics by Colorectal Cancer Screening Participation, Among Cohort Members Diagnosed With Incident Colorectal Cancer

eTable 3. Associations Between Lower Endoscopy and Colorectal Cancer Incidence Stratified by Timing of CRC Screening, Among African American Participants Eligible for Screening at Baseline Interview

eTable 4. Associations Between Lower Endoscopy and Colorectal Cancer Mortality Stratified by Timing of CRC Screening and Sex, Among African American Participants Eligible for Screening at Baseline Interview

eTable 5. Associations Between Lower Endoscopy and All-Cause Mortality Stratified by Timing of CRC Screening, Among Participants Eligible for Screening at Baseline Interview

eTable 6. Associations Between Colorectal Cancer Screening in the Interval Between Baseline and Follow-up Interview With All-Cause Mortality

eFigure. Study Design

This supplementary material has been provided by the authors to give readers additional information about their work.

© 2019 Warren Andersen S et al. *JAMA Network Open*.

eTable 1. Selected baseline characteristics by colorectal cancer screening participation, among cohort members not diagnosed with incident colorectal cancer.

	Screening modality participation at baseline interview				Follow-up Interview
**Baseline Characteristic, No. (%)**	**Never** (N=24,014)	**Ever**[a] (N=13,236)	**Colonoscopy** (N=10,316)	**Sigmoidoscopy** (N=5,909)	**Fecal occult blood test**[b] (N=9,320)
Age, median (IQR), years	55 (9)	58 (10)	58 (10)	58 (10)	57 (10)
Sex					
Men	10,105 (42.1)	4,846 (36.6)	3,660 (35.5)	2,356 (39.9)	3,308 (35.5)
Women	13,909 (57.9)	8,390 (63.4)	6,656 (64.5)	3,553 (60.1)	6,012 (64.5)
Race					
White	6,594 (27.5)	4,365 (33.0)	3,620 (35.1)	1,751 (29.6)	2,871 (30.8)
African American	16,430 (68.4)	8,229 (62.2)	6,156 (59.7)	3,874 (65.6)	6,035 (64.8)
Other	990 (4.1)	642 (4.9)	540 (5.2)	284 (4.8)	414 (4.4)
Enrollment Source					
Community Health Center	21,387 (89.1)	10,508 (79.4)	8,089 (78.4)	4,471 (75.7)	7,243 (77.7)
General Population	2,627 (10.9)	2,728 (20.6)	2,227 (21.6)	1,438 (24.3)	2,077 (22.3)
Education					
< High school	8,139 (33.9)	3,686 (27.8)	2,727 (26.4)	1,702 (28.8)	2,479 (26.6)
High school	7,679 (32.0)	3,649 (27.6)	2,878 (27.9)	1,537 (26.0)	2,613 (28.0)
> High school	8,102 (33.7)	5,805 (43.9)	4,625 (44.8)	2,621 (44.4)	4,166 (44.7)
Household income, $					
< 15,000	14,280 (59.5)	6,293 (47.5)	4,745 (46.0)	2,837 (48.0)	4,295 (46.1)
15,000-49,999	7,837 (32.6)	4,754 (35.9)	3,743 (36.3)	2,136 (36.1)	3,454 (37.1)
≥ 50,000	1,540 (6.4)	1,977 (14.9)	1,646 (16.0)	853 (14.4)	1,406 (15.1)
Insurance, yes	13,830 (57.6)	10,324 (78.0)	8,241 (79.9)	4,535 (76.7)	6,764 (72.6)
Family history of colorectal cancer	2,643 (11.0)	1,463 (11.1)	1,212 (11.7)	596 (10.1)	925 (9.9)
Obese body mass index (≥ 30.0 kg/m^2)	10,382 (43.2)	6,174 (46.6)	4,805 (46.6)	2,718 (46.0)	4,535 (48.7)
Smoking status					
Current	9,525 (39.7)	3,332 (25.2)	2,519 (24.4)	1,480 (25.0)	2,251 (24.2)
Former	5,686 (23.7)	4,157 (31.4)	3,254 (31.5)	1,911 (32.3)	2,919 (31.3)
Never	8,646 (36.0)	5,627 (42.5)	4,442 (43.1)	2,454 (41.5)	4,065 (43.6)
Non and moderate alcohol consumers	19,761 (82.3)	11,695 (88.4)	9,132 (88.5)	5,170 (87.5)	8,163 (87.6)
Physical activity, median (IQR), MET-hrs/day	16.2 (20.8)	14.9 (17.3)	14.8 (17.1)	15.1 (17.7)	15.9 (17.9)

[a] Ever screening includes colonoscopy, and sigmoidoscopy screening modalities at baseline interview.
[b] Fecal occult blood test assessment conducted during follow-up Interview.
Subjects with missing data not included in this analysis.
Screening modalities are not mutually exclusive.
Analyses include: participants eligible for colorectal cancer screening at baseline interview via age and family history criteria.

© 2019 Warren Andersen S et al. *JAMA Network Open*.

eTable 2. Selected baseline characteristics by colorectal cancer screening participation, among cohort members diagnosed with incident colorectal cancer.

	Screening modality participation at baseline interview				Follow-up Interview
**Baseline Characteristic, No. (%)**	**Never** (N=418)	**Ever** [a] (N=135)	**Colonoscopy** (N=97)	**Sigmoidoscopy** (N=69)	**Fecal occult blood test** [b] (N=141)
Age, median (IQR), years	57 (10)	59 (11)	61 (11)	60 (11)	58 (10)
Sex					
Men	171 (40.9)	63 (46.7)	42 (43.3)	37 (53.6)	55 (39.0)
Women	247 (59.1)	72 (53.3)	55 (56.7)	32 (46.4)	86 (61.0)
Race					
White	96 (23.0)	32 (23.7)	23 (23.7)	13 (18.8)	37 (26.2)
African American	307 (73.4)	101 (74.8)	74 (76.3)	54 (78.3)	100 (70.9)
Other	15 (3.6)	2 (1.5)	--	2 (2.9)	4 (2.8)
Enrollment Source					
Community Health Center	369 (88.3)	114 (84.4)	85 (87.6)	54 (78.3)	111 (78.7)
General Population	49 (11.7)	21 (15.6)	12 (12.4)	15 (21.7)	30 (21.3)
Education					
< High school	147 (35.2)	50 (37.0)	37 (38.1)	23 (33.3)	51 (36.2)
High school	129 (30.9)	39 (28.9)	28 (28.9)	19 (27.5)	32 (22.7)
> High school	138 (33.0)	46 (34.1)	32 (33.0)	27 (39.1)	55 (39.0)
Household income, $					
< 15,000	253 (60.5)	70 (51.9)	49 (50.5)	34 (49.3)	73 (51.8)
15,000-49,999	129 (30.9)	50 (37.0)	40 (41.2)	25 (36.2)	49 (34.8)
≥ 50,000	30 (7.2)	12 (8.9)	6 (6.2)	9 (13.0)	17 (12.1)
Insurance, yes	234 (56.0)	103 (76.3)	74 (76.2)	56 (81.2)	96 (68.1)
Family history of colorectal cancer	46 (11.0)	14 (10.4)	14 (14.4)	3 (4.3)	16 (11.3)
Obese body mass index (≥ 30.0 kg/m^2)	163 (39.0)	78 (57.8)	56 (57.7)	41 (59.4)	70 (49.6)
Smoking status					
Current	135 (32.3)	27 (20.0)	18 (18.6)	14 (20.3)	34 (24.1)
Former	120 (28.7)	44 (32.6)	30 (30.9)	25 (36.2)	46 (32.6)
Never	161 (38.5)	63 (46.7)	49 (50.5)	29 (42.0)	59 (41.8)
Non and moderate alcohol consumers	365 (87.3)	121 (89.6)	87 (89.7)	64 (92.8)	125 (88.7)
Physical activity, median (IQR), MET-hrs/day	16.3 (18.8)	14.3 (20.0)	15.5 (18.3)	17.0 (22.0)	20.7 (19.6)

[a] Ever screening includes colonoscopy, sigmoidoscopy screening modalities at baseline interview.
[b] Fecal occult blood test assessment conducted during follow-up Interview.
Subjects with missing data not included in this analysis.
Screening modalities are not mutually exclusive.
Analyses include: participants eligible for colorectal cancer screening at baseline interview via age and family history criteria.

© 2019 Warren Andersen S et al. *JAMA Network Open*.

eTable 3. Associations between lower endoscopy and colorectal cancer incidence stratified by timing of CRC screening, among African American participants eligible for screening at baseline interview.

Screening participation	Lower Endoscopy				Colonoscopy				Sigmoidoscopy		
	Cohort	Events	HR [a] (95%CI)		Cohort	Events	HR [a] (95%CI)		Cohort	Events	HR [a] (95%CI)
	**Colorectal Cancer Incidence**										
All participants											
Never Screened prior to baseline	16,737	307	1 (reference)		18,829	335	1 (reference)		21,159	353	1 (reference)
Ever Screened prior to baseline	8,330	101	0.59 (0.46-0.75)		6,230	74	0.65 (0.50-0.85)		3,928	54	0.70 (0.52-0.96)
Cases diagnosed prior to follow-up interview											
Never Screened prior to baseline	16,632	202	1 (reference)		18,716	222	1 (reference)		21,031	225	1 (reference)
Ever Screened prior to baseline	8,288	59	0.54 (0.39-0.74)		6,196	40	0.55 (0.38-0.79)		3,909	35	0.73 (0.49-1.08)
Cases diagnosed after follow-up interview [b]											
Never Screened prior to follow-up interview	5,658	70	1 (reference)		6,496	77	1 (reference)		9,619	101	1 (reference)
Screened prior to baseline only	2,553	25	0.70 (0.44-1.12)		1,946	20	0.81 (0.49-1.33)		2,083	15	0.60 (0.35-1.03)
Screened between baseline and follow-up interview only	3,161	21	0.47 (0.29-0.77)		3,653	21	0.42 (0.26-0.69)		1,292	7	0.46 (0.21-0.98)
Screened both prior to baseline and between baseline and follow-up interview	3,173	17	0.36 (0.21-0.62)		2,440	14	0.43 (0.24-0.77)		531	4	0.60 (0.22-1.65)

[a] Models are adjusted for enrollment source, sex, health insurance status, smoking status, education, income, alcohol intake, body mass index, and family history of colorectal cancer. Statistical models include variables for unknown screening status at baseline or follow-up interview (results not displayed).
[b] Participants included in the analysis must have completed follow-up interview.
Analyses include: participants eligible for colorectal cancer screening at baseline interview via age and family history criteria.

© 2019 Warren Andersen S et al. *JAMA Network Open*.

eTable 4. Associations between lower endoscopy and colorectal cancer mortality stratified by timing of CRC screening and sex, among African American participants eligible for screening at baseline interview.

Screening participation	Lower Endoscopy				Colonoscopy				Sigmoidoscopy		
	Cohort	Deaths	HR [a] (95%CI)		Cohort	Deaths	HR [a] (95%CI)		Cohort	Deaths	HR [a] (95%CI)
	Women										
All participants											
Never Screened prior to baseline	9,525	61	1 (reference)		10,798	64	1 (reference)		12,464	69	1 (reference)
Ever Screened prior to baseline	5,321	13	0.35 (0.19-0.65)		4,045	10	0.44 (0.22-0.86)		2,390	5	0.29 (0.11-0.81)
Participants without follow-up interview data											
Never Screened prior to baseline	3,020	31	1 (reference)		3,375	34	1 (reference)		3,729	32	1 (reference)
Ever Screened prior to baseline	1,343	5	0.30 (0.11-0.88)		987	2	0.22 (0.05-0.92)		641	4	0.56 (0.17-1.85)
Participants with follow-up interview data [b]											
Never Screened prior to follow-up interview	3,650	19	1 (reference)		4,229	19	1 (reference)		6,457	31	1 (reference)
Screened prior to baseline only	1,725	6	0.60 (0.24-1.52)		1,309	6	0.98 (0.38-2.48)		1,419	1	0.13 (0.02-0.95)
Screened between baseline and follow-up interview only	2,247	10	0.76 (0.35-1.65)		2,588	10	0.78 (0.36-1.69)		876	4	0.85 (0.30-2.42)
Screened both prior to baseline and between baseline and follow-up interview	2,253	2	0.15 (0.04-0.67)		1,749	2	0.25 (0.06-1.11)		330	0	--
	Men										
All participants											
Never Screened prior to baseline	7,175	38	1 (reference)		7,987	43	1 (reference)		8,651	44	1 (reference)
Ever Screened prior to baseline	2,990	17	0.86 (0.47-1.57)		2,173	12	0.89 (0.46-1.73)		1,526	11	1.09 (0.55-2.16)
Participants without follow-up interview data											
Never Screened prior to baseline	3,841	22	1 (reference)		4,232	24	1 (reference)		4,430	27	1 (reference)
Ever Screened prior to baseline	1,228	11	1.33 (0.62-2.83)		836	9	1.75 (0.79-3.86)		652	6	1.21 (0.49-3.01)
Participants with follow-up interview data [b]											
Never Screened prior to follow-up interview	2,010	4	1 (reference)		2,273	7	1 (reference)		3,194	9	1 (reference)
Screened prior to baseline only	829	2	0.93 (0.16-5.25)		637	0	--		672	3	1.17 (0.31-4.49)

© 2019 Warren Andersen S et al. *JAMA Network Open*.

Screened between baseline and follow-up interview only	973	9	4.28 (1.29-14.26)		1,127	9	2.37 (0.85-6.58)		439	5	3.15 (1.04-9.60)
Screened both prior to baseline and between baseline and follow-up interview	933	4	1.61 (0.37-6.92)		700	3	1.10 (0.26-4.55)		202	2	2.39 (0.48-12.00)

[a] Models are adjusted for enrollment source, health insurance status, smoking status, education, income, alcohol intake, body mass index, and family history of colorectal cancer. Statistical models include variables for unknown screening status at baseline or follow-up interview (results not displayed).
[b] Participants included in the analysis must have completed follow-up interview.
Analyses include: participants eligible for colorectal cancer screening at baseline interview via age and family history criteria.

© 2019 Warren Andersen S et al. *JAMA Network Open*.

eTable 5. Associations between lower endoscopy and all-cause mortality stratified by timing of CRC screening, among participants eligible for screening at baseline interview.

Screening participation	Lower Endoscopy				Colonoscopy				Sigmoidoscopy		
	Cohort	Deaths	HR [a] (95%CI)		Cohort	Deaths	HR [a] (95%CI)		Cohort	Deaths	HR [a] (95%CI)
All participants											
Never Screened prior to baseline	24,432	5,878	1 (reference)		27,382	6,643	1 (reference)		31,841	7,501	1 (reference)
Ever Screened prior to baseline	13,371	3,114	1.02 (0.97-1.06)		10,413	2,347	1.02 (0.97-1.07)		5,978	1,498	1.04 (0.98-1.10)
Participants without follow-up interview data											
Never Screened prior to baseline	9,265	3,551	1 (reference)		10,224	3,988	1 (reference)		11,195	4,379	1 (reference)
Ever Screened prior to baseline	3,637	1,635	1.13 (1.06-1.20)		2,676	1,200	1.14 (1.06-1.22)		1,732	817	1.10 (1.02-1.19)
Participants with follow-up interview data [b]											
Never Screened prior to follow-up interview	9,021	1,394	1 (reference)		10,276	1,617	1 (reference)		16,057	2,311	1 (reference)
Screened prior to baseline only	4,465	754	1.05 (0.95-1.14)		3,566	572	1.02 (0.93-1.13)		3,490	564	1.02 (0.93-1.12)
Screened between baseline and follow-up interview only	4,890	673	0.89 (0.81-0.97)		5,633	766	0.86 (0.78-0.93)		1,798	314	1.01 (0.90-1.14)
Screened both prior to baseline and between baseline and follow-up interview	5,269	725	0.87 (0.80-0.96)		4,171	575	0.92 (0.83-1.01)		756	117	0.88 (0.73-1.06)

[a] Models are adjusted for enrollment source, race, sex, health insurance status, smoking status, education, income, alcohol intake, body mass index, and family history of colorectal cancer. Statistical models include variables for unknown screening status at baseline or follow-up interview (results not displayed).
[b] Participants included in the analysis must have completed follow-up interview.
Analyses include: participants eligible for colorectal cancer screening at baseline interview via age and family history criteria.

© 2019 Warren Andersen S et al. *JAMA Network Open*.

eTable 6. Associations between colorectal cancer screening in the interval between baseline and follow-up interview with all-cause mortality.

Screening participation	Any Screening				Colonoscopy				Sigmoidoscopy				Fecal Occult Blood Test		
	Cohort	Deaths	HR [a] (95%CI)		Cohort	Deaths	HR [a] (95%CI)		Cohort	Deaths	HR [a] (95%CI)		Cohort	Deaths	HR [a] (95%CI)
No screening between baseline and follow-up interview	13,975	1,902	1 (reference)		19,680	2,706	1 (reference)		26,839	3,478	1 (reference)		18,986	2,463	1 (reference)
Screened between baseline and follow-up interview	18,042	2,369	0.91 (0.85-0.97)		12,533	1,577	0.91 (0.85-0.97)		3,263	512	1.03 (0.94-1.13)		12,119	1,670	0.98 (0.92-1.04)

[a] Models are adjusted for enrollment source, race, sex, health insurance status, smoking status, education, income, alcohol intake, body mass index, and family history of colorectal cancer.
Statistical models include variables for unknown screening status at baseline or follow-up interview (results not displayed).
Analyses include: participants eligible for colorectal cancer screening at follow-up interview via age and family history criteria, and who completed the follow-up interview.

© 2019 Warren Andersen S et al. *JAMA Network Open*.

eFigure. Study design.

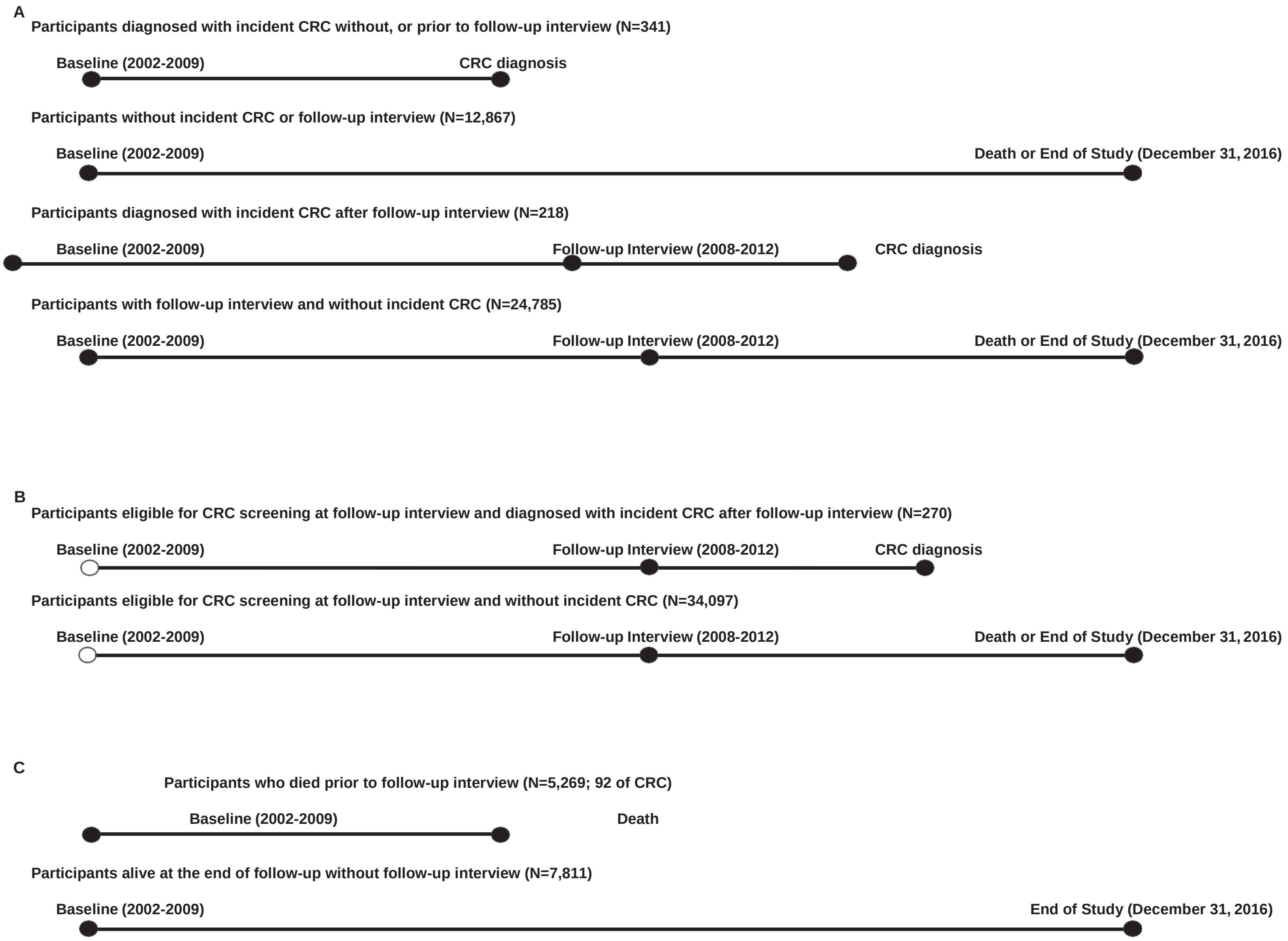

© 2019 Warren Andersen S et al. *JAMA Network Open*.

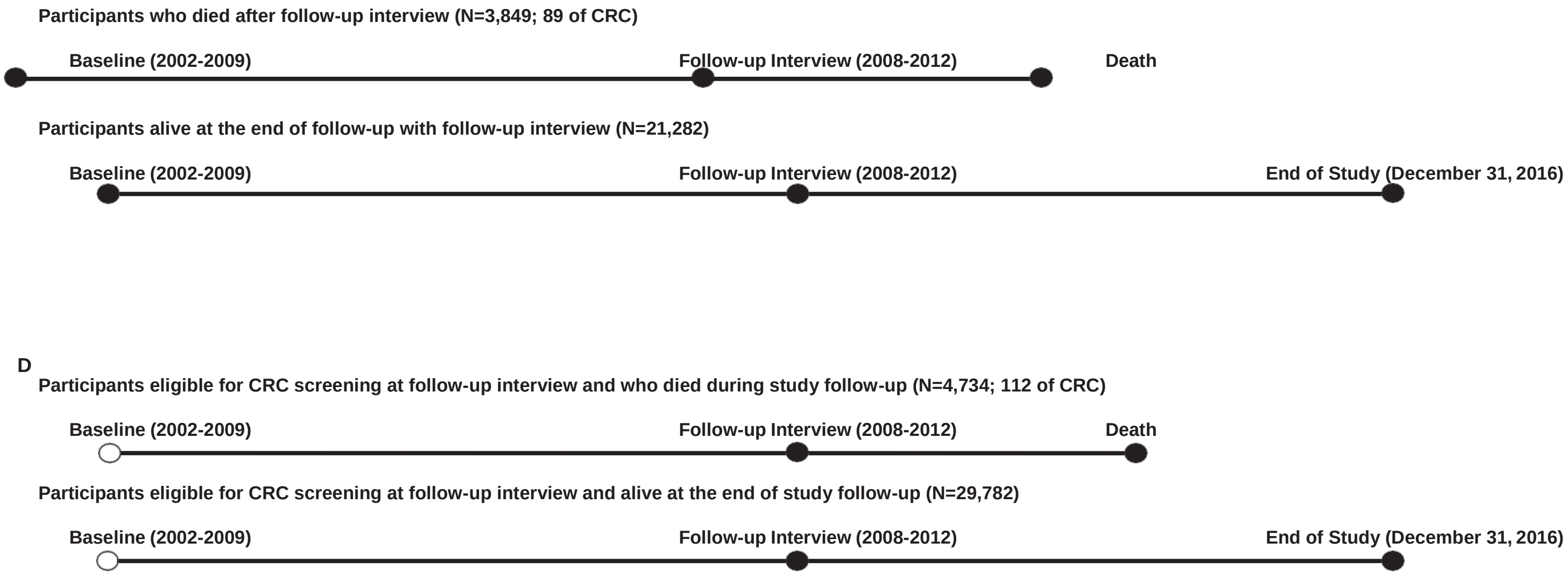

Timeline of the study for CRC incidence and mortality analyses stratified by eligibility for CRC screening, availability of follow-up interview information and vital status. Top portion of the Figure refers to CRC incidence analysis A) Participants eligible for CRC screening at baseline study entry and B) Participants eligible for CRC screening at follow-up interview. Bottom portion of the Figure described study design for mortality analysis C) Participants eligible for CRC screening at baseline study entry and D) Participants eligible for CRC screening at follow-up interview. CRC, colorectal cancer.

© 2019 Warren Andersen S et al. *JAMA Network Open*.
